# Penetration Enhancers in Ocular Drug Delivery

**DOI:** 10.3390/pharmaceutics11070321

**Published:** 2019-07-09

**Authors:** Roman V. Moiseev, Peter W. J. Morrison, Fraser Steele, Vitaliy V. Khutoryanskiy

**Affiliations:** 1 Reading School of Pharmacy, University of Reading, Whiteknights, P.O. Box 224, Reading RG66AD, UK; 2 MC2 Therapeutics, James House, Emlyn Lane, Leatherhead KT22 7EP, UK

**Keywords:** ocular drug delivery, cornea, penetration enhancers, ocular conditions, ophthalmology

## Abstract

There are more than 100 recognized disorders of the eye. This makes the development of advanced ocular formulations an important topic in pharmaceutical science. One of the ways to improve drug delivery to the eye is the use of penetration enhancers. These are defined as compounds capable of enhancing drug permeability across ocular membranes. This review paper provides an overview of anatomical and physiological features of the eye and discusses some common ophthalmological conditions and permeability of ocular membranes. The review also presents the analysis of literature on the use of penetration-enhancing compounds (cyclodextrins, chelating agents, crown ethers, bile acids and bile salts, cell-penetrating peptides, and other amphiphilic compounds) in ocular drug delivery, describing their properties and modes of action.

## 1. Introduction

According to the World Health Organization, the number of people who live with some form of distance or near vision impairment is about 1.3 billion worldwide [1]. This problem is very important because approximately 80% of external input of information delivered to the brain is processed by the visual pathway [2]. There were many methods and improvements of ocular drug delivery developed over the last decades exploring more effective treatments for different ocular diseases. Nevertheless, this field of medicine remains one of the most challenging. 

The preferred method of ocular drug delivery is via topical application due to ease of access to the eye and the non-invasive nature of this administration route [3]. Self-medication by this means is achievable by most people that are not limited by dexterity issues or conditions affecting mental ability. Administration by a helper eliminates these potential difficulties. Ocular drug penetration is possible via the transcellular pathway, i.e., into and through cells, or the paracellular route, i.e., between cells, or a combination of both pathways [4]. 

Drug penetration enhancement can be achieved by inclusion of agents capable of modifying the tear film, mucous layer, and ocular membranes in a drug formulation [5]. A further strategy for enhancing drug penetration into the eye can be achieved by energy-driven means, where a small electrical current is used (iontophoresis) [6] or ultrasound is employed to drive the drug to enter the ocular tissues [7,8,9]. The use of microneedles for enhanced ocular drug delivery is another emerging area of research; however, this could be considered somewhat invasive and inconvenient [10,11,12].

Penetration enhancers are compounds that are able to enhance drug delivery across otherwise impermeable or limited permeability membranes such as the cornea, acting predominantly on the epithelia [5]. The use of penetration enhancers in transdermal applications is a well-established approach to facilitate drug delivery across the skin and this topic was covered in a number of excellent reviews [13,14]. Ocular drug delivery is still lacking good understanding and analysis of the effects of various penetration enhancers. It is, however, already established that penetration enhancers in ocular drug delivery facilitate delivery of active pharmaceutical ingredients through three main mechanisms or their combination [15,16]:

1. Altering tear film stability and the mucous layer at the ocular surface [17,18];
2. Modifying membrane components such as lipid bilayers of associated epithelial cells [19];
3. Loosening epithelial tight junctions [4,17].

Penetration-enhancing excipients for use in ocular formulations should ideally have the following characteristics: they should be non-toxic and non-irritating, efficacious at low concentrations, fast-acting, and their effect should be reversible [20,21]. 

This paper provides an overview of anatomy and physiology of the eye, discusses some common ophthalmological conditions, and presents an analysis of literature on the use of penetration enhancers in ocular drug delivery.

## 2. Ocular Anatomy and Physiology 

**Figure 1:**
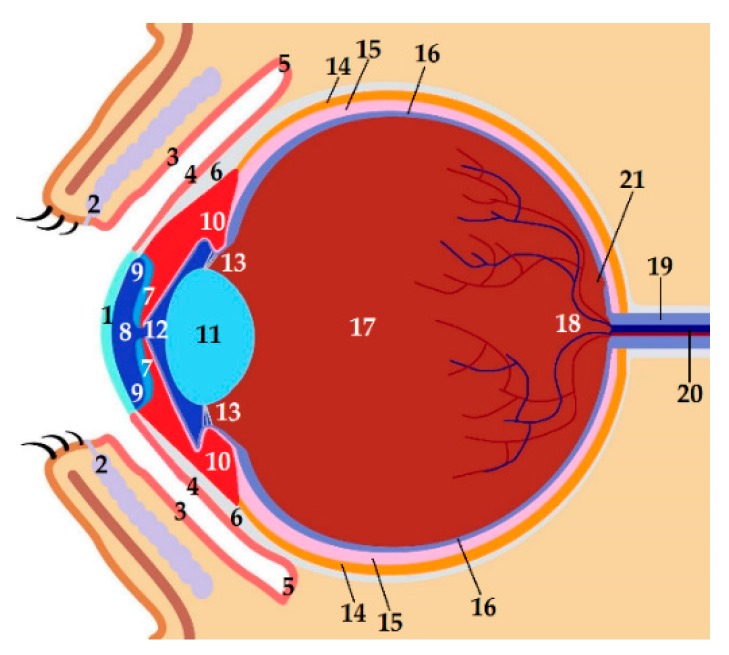
Anatomy of the human eye: 1—cornea; 2—meibomian glands; 3—palpebral conjunctiva; 4—bulbar conjunctiva; 5—conjunctival fornix; 6—sclera; 7—iris; 8—anterior chamber; 9—iridocorneal angle; 10—ciliary body; 11—lens; 12—posterior chamber; 13—suspensory ligament; 14—choroid; 15—retinal pigmented epithelium; 16—retina; 17—vitreous body; 18—optic disc; 19—optic nerve; 20—central artery and vein of the retina; 21—fovea.

The visual system is a sensory unit helping us to understand our environment, comprising a receptor (retina), sensory pathway (optic tract), and brain center (primary visual cortex). Each eyeball is embedded within the orbit, which is a pear-shaped cavity physically protecting the eye [1,22]. Adipose tissue surrounds the eye within the orbital cavity and helps to cushion the eye. Other accessory organs include lacrimal glands, eyebrows, eyelids, eyelashes, and muscles of the eye. The human eye is a complex organ comprising three different coats or layers, enclosing various anatomical structures as shown in Figure 1. Below, we briefly consider some specific tissues in the eye.

### 2.1. The Outermost Layer (Tunica Fibrosa Oculi)

**Figure 2:**
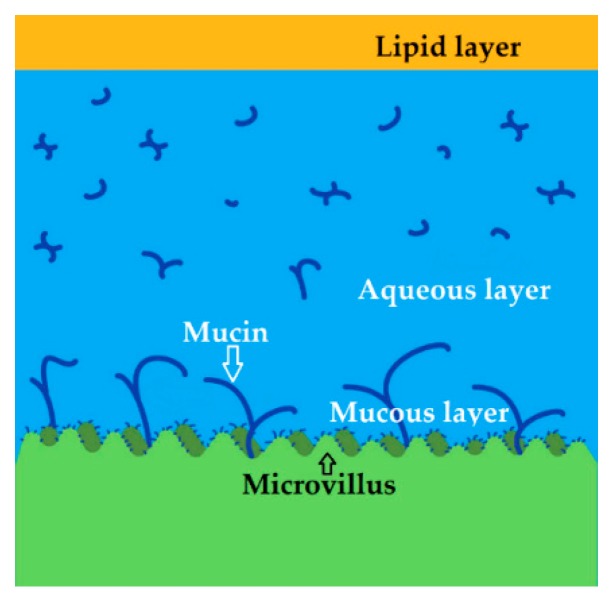
The tear film consists of the outer lipid layer, middle aqueous layer, and mucous layer.

The fibrous tunic helps maintain the spherical shape of the eyeball and consists of the cornea and sclera. It is well known that less than 5% of the topically applied drug penetrates through the cornea [23]. However, the most accessible regions for drug application are the conjunctiva, sclera, and cornea. The cornea is an avascular, transparent, highly innervated structure, whose average diameter is ~11.5 mm vertically and ~12 mm horizontally. The average thickness of this structure is ~540 μm in the central part, and it is thicker toward the periphery [22]. The refractive power of this structure in the eye is roughly 42 diopters. The cornea is covered with the tear fluid, forming a film that protects its surface from dust and other particles. This film consists of three layers as shown in Figure 2: the outer lipid layer (delivered to the lid margins from the meibomian glands located within the tarsal plate) [24], middle aqueous layer (produced by the lacrimal gland, containing free lipid and soluble mucin), and mucous layer (which is mainly secreted by the goblet cells located within the conjunctiva as single cells with the highest density of those in the conjunctiva of the inferior eyelid) [25]. 

**Figure 3:**
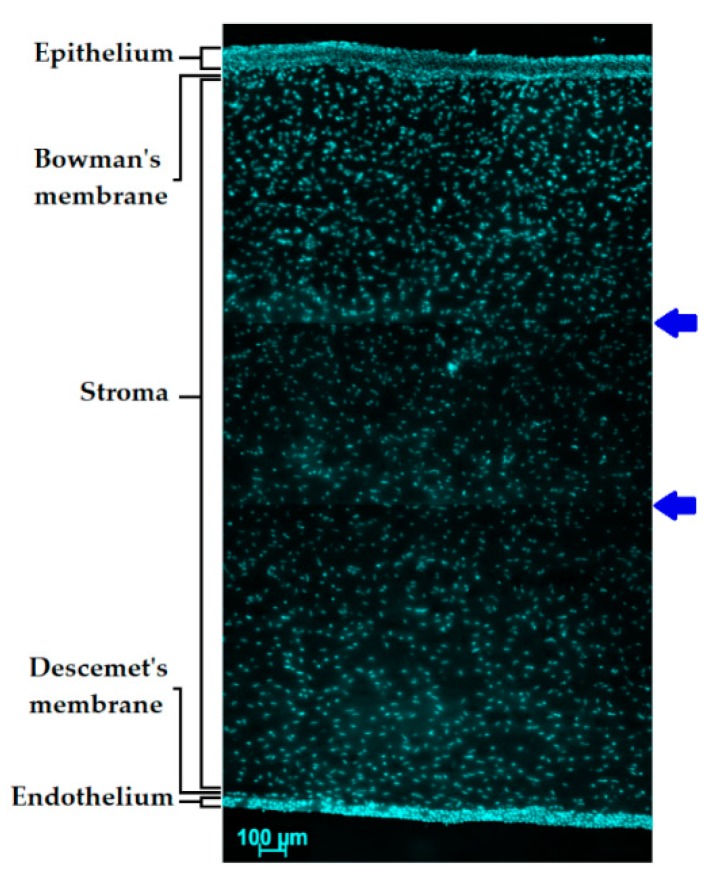
Micrograph demonstrating a cross-section of the multilayered structure of porcine cornea. Scale bar = 100 µm. Please note that this micrograph is a combination of three images stitched together using Inkscape 0.92.4 software due to the restrictions of the microscope camera field of view posing restrictions to the image in view. The arrows indicate stitches between images.

The mucous layer is in contact with the corneal epithelium and is anchored via microvilli of the superficial epithelium cells. Apart from the tear film, there is another factor hampering ocular drug delivery—the structure of the cornea (Figure 3). The cornea consists of six different layers (epithelium, Bowman’s membrane, stroma, Dua’s layer, Descemet’s membrane, and endothelium). The external layer of the cornea is the epithelium. It is relatively thin and is about 100 µm in the periphery of the cornea and approximately 50 µm in its center.

The main feature of this layer is the ability to regenerate non-keratinized stratified squamous epithelium. The high lipophilicity of the epithelium allows permeability to up to 90% of lipophilic small drug molecules and only about 10% of hydrophilic molecules. The cells of this layer have Ca^2+^-dependent membrane adherent regions; tight junctions are formed between the zonula occludens, zonula adherens, and desmosomes [5]. The epithelium protects the eye against ultraviolet radiation (UVR) by means of a high concentration of tryptophan residues and ascorbate that absorb UVR [26]. The next layer that is placed right underneath the epithelium is the Bowman’s membrane that is also called the anterior limiting membrane. Bowman’s layer is acellular and composed primarily of collagen. The anterior limiting membrane reaches from 8 µm to 14 µm in thickness. The middle corneal layer is the stroma, also known as substantia propria due to the fact that it makes up to 90% of the total thickness of the cornea. It is composed of about 80% of water and collagen, proteins, mucopolysaccharides, and proteoglycans [5]. In 2013, Dua et al. reported a discovery of an additional corneal layer that was not detected previously [27]. According to Dua et al., this layer is about 15 µm in thickness and is located between the corneal stroma and the fifth corneal layer, Descemet’s membrane. This is also the posterior limiting membrane of the cornea. This membrane is a ~6-µm-thick basement of the endothelium cells. The innermost layer of the cornea is known as the endothelium and plays the role of a pump, providing the cornea with the correct hydration to ensure transparency of this tissue. The clarity of the cornea depends on an ordered lamellar collagen structure and relative dehydration which requires endothelial cell density of at least 1000 cells/mm^2^. According to the literature, there is a loss of the human cornea mean endothelial cell density in normal eye by approximately 0.6% per year [28]. The endothelium is ~5 µm in thickness and consists of a monolayer of squamous or low cuboidal cells [5]. 

**Figure 4:**
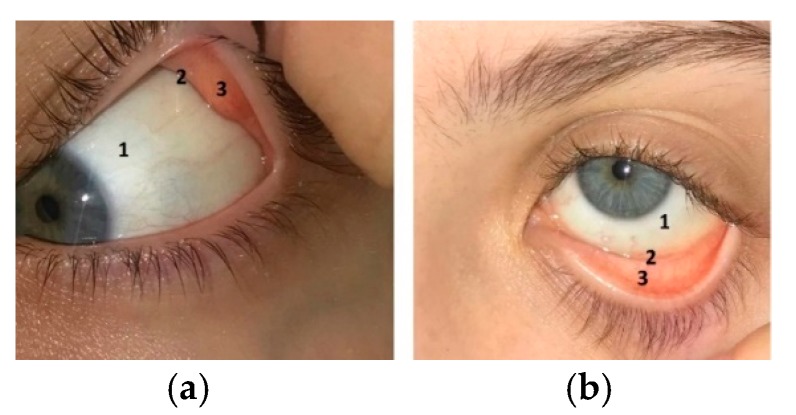
Three portions of conjunctiva: (**a**) 1—bulbar conjunctiva; 2—superior conjunctival fornix; 3—palpebral conjunctiva of the upper lid; (**b**) 1—bulbar conjunctiva; 2—inferior conjunctival fornix; 3—palpebral conjunctiva of the lower lid.

Another layer providing the eyeball with protection is called the conjunctiva, of which all three portions are shown in Figure 4. The palpebral conjunctiva has blood vessels and covers the posterior surface of the upper and lower lids. The conjunctiva comprises two or more layers of isoprismatic to highly prismatic epithelial cells. The bulbar conjunctiva is an avascular slightly mobile layer that starts from the upper and lower fornices and lies over the sclera up to the cornea region. This anatomical coat consists of stratified non-keratinized epithelial cells [29]. The superior and inferior fornices form the conjunctival sac, which can act as a reservoir for instilled medicine or the placement of a drug-loaded ocular insert [30,31]. According to the literature, the estimated total area of the human conjunctival sac is approximately 16 cm^2^ [24]. Figure 4 clearly demonstrates all three portions of the conjunctiva.

The tough outer layer of the eye globe is called the sclera. This is covered by the episcleral, a loose connective tissue layer. In the anterior part of the eye, the sclera lies up to the corneal limbus, and posteriorly to the optic nerve. This layer comprises collagen, proteoglycans, elastin, and glycoproteins. These collagen bundles are not uniformly oriented, which results in the opaque but mechanically strong nature of the sclera. The sclera itself is pierced by a number of blood vessels, while being effectively avascular itself [32].

### 2.2. Eye Chambers, Iris, Ciliary Body, and Lens

One of the first intraocular structures that can be partially seen, due to the corneal transparency, is the anterior chamber of the eye. This is a space between the cornea’s endothelium and anterior surface of the iris that is filled with aqueous humor. The latter is an optically clear fluid resembling blood plasma. The aqueous humor helps maintain appropriate intraocular pressure (IOP), and provides the cornea, trabecular meshwork, and lens with nutrition and oxygen, while removing metabolic wastes, as well as delivering molecules of neurotransmitters [33]. 

**Figure 5:**
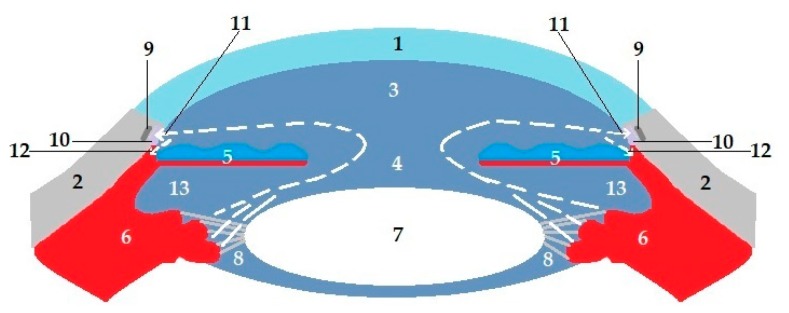
Pathways of aqueous humor outflow are indicated with arrows. 1—cornea; 2—sclera; 3—anterior chamber; 4—pupil; 5—iris; 6—ciliary body; 7—lens; 8—suspensory ligament; 9—Schlemm’s canal; 10—trabecular meshwork; 11—trabecular route; 12—uveoscleral route; 13—posterior chamber.

This fluid is formed and delivered to the posterior chamber by nonpigmented cells of the epithelia via ciliary processes [34]. Then, it flows through the pupil into the anterior chamber where two ways of outflow are found (Figure 5): (1) the conventional route is the primary outflow, where the aqueous humor is drained through the trabecular meshwork located at the anterior chamber angle (iridocorneal angle) to the Schlemm’s canal (circular tube located in the limbus, 1-mm-wide region of merging epithelium of cornea and sclera), which delivers it directly into aqueous veins [35]; (2) uveoscleral outflow is provided by means of the iris root and the ciliary body face, where the aqueous humor crosses between fibers of the muscle into the supraciliary and suprachoroidal spaces and is collected by the choroidal blood circulation. The proportion of aqueous outflow via each route could be different according to the age and disease [36]. The aqueous humor consists of inorganic and organic ions, carbohydrates, glutathione, urea, amino acids, proteins, oxygen, carbon dioxide, and water. According to the literature, the concentration of Na^+^ in the aqueous humor is almost the same as in the blood plasma, while the concentration of proteins is 200 times lower compared to plasma levels, and ascorbate’s concentration is 20 to 50 times higher [33].

The anterior chamber houses the iris, which separates anterior and posterior segments. Being attached to the anterior part of the ciliary body from the periphery, this anatomical structure has an aperture in its center that is called the pupil, the diameter of which can be regulated by the sphincter and dilator muscles located within the iris stroma (vascular connective tissue) to control the amount of light transmitting into the eye. At the posterior part of the iris, there are two epithelial layers—the anterior layer is slightly pigmented, while the posterior one has a high density of pigmentation and faces the posterior chamber. Just behind the iris lies another part of the uveal tract called the ciliary body. As already mentioned, production of the aqueous humor and uveoscleral outflow are among its functions. Additionally, the ciliary body is responsible for hyaluronate production and its release into the vitreous body, as well as for being a part of the accommodation mechanism due to the circular muscle fibers within the ciliary body. Also, this structure of the eye plays an important role in maintaining the blood–aqueous barrier [37]. 

The capsular bag is situated between the iris and the vitreous body and encapsulates the crystalline lens, which is a transparent biconvex avascular structure enclosed in the elastic capsule composed of collagen and that is attached to the ciliary body with the help of zonular fibers (the zonule of Zinn). Interestingly, the thickness of the anterior part of the capsular bag grows from 12–15 µm to 21 µm throughout life. Conversely, the thickness of the posterior part remains at about 2 µm. It is well known that the lens is the second most powerful refractive structure of the eye (after the cornea). It helps refract incoming light and focuses it onto the retina. When light is focused at a short focal distance, the suspensory ligaments are not under tension due to contraction of the ciliary muscle; thus, the lens thickens and results in higher refractive power. At the same time, distance vision requires the ciliary muscle to relax and, hence, to tense the lens-supportive apparatus which flattens the lens. Regarding the microscopic structure of the lens, it is composed of two types of cells. The cuboidal lens epithelial cells form a single layer that lines the anterior hemisphere, enlarging in the equator region and forming the lens “bow”. The cells in this region function as stem cells for the lens. The bulk of the lens consists of lens fibers that differentiate to the point where no lens nucleus remains. These fiber cells extend in concentric layers from the anterior lens pole to the posterior one, where junctions at the terminus of the cells form the lens sutures [38]. The transparency and refractive properties of the lens are due to the uniform concentration gradient of the crystallins (α-, β-, γ-crystallins) that represent about 90% of the water-soluble proteins [39]. 

The third chamber of the eye is called the vitreous body, which is positioned at the back of the eyeball between the lens and the retina. It is a clear, gel-like substance occupying the main volume of the eye and is composed of long fine collagen fibers, proteins (over 80% of those are albumin and immunoglobulins), fibrillins, fibulins, agrin, opticin, pigment epithelium-derived factor, leucine-rich alpha-2 glycoprotein, thrombospondins, hyaluronic acid, polysaccharides, ascorbic acid, and water (about 98% of the whole volume). The vitreous body has no circulatory flow; hence, molecular movements are driven mostly by diffusion. Although the vitreous body is a remarkably stable structure, there is a gradual tendency for the gel to collapse in the course of a lifetime. This is commonly believed to be a result of degradation or alteration of the collagen fiber network. Moreover, the volume of the vitreous gel is about 4 mL at the age of 20 years, starting to gradually decrease after 40 years to less than 2.5 mL at 80 years [40,41,42].

### 2.3. Choroidea and Retina

The choroid is a vascular layer located between the sclera and Bruch’s membrane (BM) extending from the ora serrata (region of the anterior edge of the retina) to the optic nerve. The choroid coat includes five distinct regions: (1) BM—thin, acellular extracellular matrix positioned right under the retina which acts like a filter to the retina; (2) the choriocapillaris (provides the outer retina with oxygen and nutrients); (3) layer with small and medium-sized vessels; (4) layer with large vessels; and (5) the suprachoroid. Apart from BM and the layer with choriocapillaris, the remainder of the choroidea, the stroma, is populated with melanocytes, connective tissue elements (including fibroblasts, mastocytes, elastic, and collagen fibrils), blood vessels, macrophages, dendritic cells, lymphocytes, nonvascular smooth muscle cells, intrinsic neurons, and nerve fibers associated with vessels [43,44,45].

The retina is a complex transparent tissue of ~0.55 mm at the edges of the fovea and of ~0.13 mm at the center (umbo). This innermost layer of the eye includes a nonsensory part and an optic part that are divided by the ora serrata. The anterior part does not provide any sensory function and covers the ciliary body and iris with bilaminar epithelium. The posterior portion of the retina lies from the ora serrata to the optic disc being attached only at these two locations. Interestingly, there is an area on the optic retina, roughly 5.5 mm in diameter, which is centered about 4 mm temporal to and 0.8 mm inferior to the center of the optic disc that is called macula (macula lutea or central retina) and which has xanthophyll and two or more layers of ganglion cells. Moreover, few regions can be distinguished within this part of the retina: (1) the fovea (fovea centralis) is a depression in the center of the macular area (~1.5 mm in diameter); (2) the foveola is a central floor of the fovea, which is approximately 0.35 mm in diameter; and (3) the umbo (central depression of the foveola). The optic part of the retina is composed of the retinal pigmented epithelium (RPE; the outer layer) and the cerebral layer (inner). RPE supplies the neurosensory layer with nutrition, as well as removes metabolic products. Additionally, it plays a key role in maintaining the blood–retina barrier (BRB) separating the neurosensory retina from the circulating blood. The inner retina comprises photoreceptors (approximately 120 million rods and 6–7 million cones that constitute the first neuron), bipolar cells (the second neuron), and multipolar ganglion cells (the third neuron), along with horizontal and amacrine cells. Interestingly, rod cells are responsible for peripheral vision acting at low light levels using the photosensitive molecule (rhodopsin) in visual phototransduction. In contrast, three types of cone cells (according to the color perception: red, blue, and green) are located only within the fovea area and are 30- to 100-fold less sensitive compared to rod cells, requiring the simultaneous activation of tens to hundreds of visual pigment molecules (that are homologous or even identical to those found in rods) to generate a detectable response resulting in their activity at relatively high light levels, thereby providing central vision and the ability to distinguish colors. Therefore, the retina is the site of transformation of light energy into a neural signal that is transmitted via the optic nerve to the visual cortex to be analyzed by the brain helping to “see” the environment [29,46,47]. 

## 3. Ocular Conditions

There are more than 100 recognized disorders of the eye [22]. Therefore, only some of the most common disorders or the conditions that could potentially benefit from the development of advanced topical formulations with enhanced drug permeability are briefly discussed here. Most frequent diseases of the cornea include keratitis, keratoconus, and dry eye syndrome. 

Keratitis is a condition when the patient’s cornea becomes inflamed. A wide range of germs could lead to this inflammatory condition, but the most common are *Staphylococcus aureus*, *Staphylococcus epidermidis*, *Streptococcus pneumoniae*, *Pseudomonas aeruginosa*, *Neisseria gonorrhoeae*, *Haemophilus*, herpes simplex and varicella zoster viruses, *Fusarium*, *Aspergillus*, *Candida*, and acanthamoeba. Patients typically complain of pain, foreign body sensation, photophobia, redness of the eye, tearing, discharge (sometimes purulent), and decreased visual acuity. Although this condition is mostly quite painful for the patient and requires urgent treatment, sometimes it can be almost asymptomatic (for instance, if caused by acanthamoeba). 

Antibacterial drops combined with subconjunctival injections in severe cases are the choices of therapy in patients with keratitis. According to the most recent clinical recommendations [48], two main schemes are used to treat bacterial keratitis depending on the pathogen and the response to its therapy. The monotherapy includes the use of antibacterial eye drops consisting of fluoroquinolones (levofloxacin, moxifloxacin, gatifloxacin, besifloxacin, ofloxacin, or ciprofloxacin) that cover both Gram-positive (*Staphylococcus aureus*, *Staphylococcus epidermidis*, *Streptococcus pneumoniae*) and Gram-negative (*Pseudomonas aeruginosa*, *Neisseria gonorrhoeae*, *Haemophilus*) bacteria. However, these medications should be used with caution due to the possible resistance of some organisms, which can be noticed by controlling the clinical response. Therefore, there is a second treatment scheme, which combines fluoroquinolone eye drops and solutions for a topical or subconjunctival route that comprise other groups of antibiotics including cephalosporin (cefazolin, ceftazidime), aminoglycosides (gentamicin or tobramycin), penicillins (penicillin G, methicillin, or piperacillin), and glycopeptide antibiotics (vancomycin). For instance, cefazolin is commonly used for *Pseudomonas aeruginosa* infection. On the other hand, ceftazidime is effective against resistant forms of *Pseudomonas aeruginosa*. Vancomycin has efficacy against methicillin-resistant staphylococci, as well as against other Gram-positives. Additionally, corticosteroids (dexamethasone, prednisolone) can be used topically in terms of reducing pain and inflammation in bacterial keratitis but with caution, keeping in mind they inhibit re-epithelialization of the cornea [49,50]. Treatment with steroids should be avoided, when keratitis is induced by fungi or acanthamoeba, as well as in cases of any doubts regarding the effectiveness of the antimicrobial regimen. 

Nonsteroidal anti-inflammatory drugs (NSAIDs) also may be recommended for reducing symptoms of inflammation only in the case of efficient antimicrobial therapy. In addition, preservative-free lubricants may be used for promoting healing and cycloplegic agents (cyclopentolate) to reduce discomfort associated with spasm of the ciliary body, as well as for decreasing formation of synechiae [48]. 

Topical usage of antiviral eye drops (acyclovir) is the core of treatment in cases of herpes simplex keratitis. Eye drops with trifluorothymidine or ganciclovir can be prescribed instead of acyclovir in cases of resistance to therapy. Also, prednisolone acetate- or dexamethasone-including eye drops are used to reduce the inflammation, albeit with the caveat regarding epithelial damage discussed above. In terms of preventing virus reactivation, the administration of oral antiviral agents is preferred. Conversely, in patients with keratitis induced by varicella zoster virus (herpes zoster ophthalmicus), Dworkin et al. [51] reported that topical antiviral therapy is ineffective. Hence, systemic usage of antiviral drugs is the choice of treatment. Additionally, cycloplegic eye drops (cyclopentolate) are used for preventing ciliary spasm that can be associated with this pathological condition. Topical preservative-free lubricants such as hydroxypropyl methylcellulose (and others) can also be added to the treatment [48]. 

Fungal keratitis should be generally treated with topical antifungal formulations that can be divided into a few groups depending on their mode of action: polyenes (amphotericin B, natamycin), imidazoles (clotrimazole, miconazole, econazole, ketoconazole), triazoles (itraconazole, fluconazole, voriconazole), pyrimidines (flucytosine), and echinocandins (micafungin, caspofungin) [49]. Systemic antifungal drugs can be recommended in severe cases involving deep stromal layers with a high risk of perforation of the cornea. Additionally, topical cycloplegic drugs can be used. The keratitis caused by acanthamoeba should be treated with topical anti-amoebic formulations [48]. Of note, the aim of this treatment is to eradicate amoebic cysts from the cornea. The first-line therapy includes biguanide agents (polyhexamethylene biguanide (PHMB), chlorhexidine) that are cystidical (active against cysts). The aromatic diamidines (propamidine, hexamidine) belong to the second-line medications providing more variable activity, along with being cystidical. In contrast, aminoglycosides (neomycin, paromomycin) or azole agents (clotrimazole, fluconazole, ketoconazole, miconazole) are almost not cystidical, being active against trophozoites [49]. However, they can be used in addition to first- or second-line therapy. According to the literature, adenylate cyclase can be administered topically to promote the conversion of cysts into a protozoal state [48]. 

Keratoconus is defined by Kanski and Bowling as a progressive disorder in which thinning of the central or paracentral stroma of the cornea occurs, accompanied by apical protrusion and irregular astigmatism [22]. It affects 0.05–5% of the population [48]. Patient complaints include blurred vision, mild photophobia, and increased vision impairment due to progressive myopia and astigmatism. The cause of this condition is still unknown, but some authors refer to such factors as combination of repeated trauma and existing abnormalities of the stroma. Additionally, in some cases, keratoconus presents in patients with other ocular and systemic conditions including, for example, disorders of the connective tissue. One widely used method for treatment of this condition is a corneal collagen cross-linking, which leads to stabilization of ectasia by using riboflavin eye drops and subsequent exposure to ultraviolet-A light. Also, implantation of the ring segment within the cornea can be used as an addition to the cross-linking procedure. In severe cases, keratoplasty (corneal transplantation) is a choice of treatment [22]. 

Dry eye disease (keratoconjunctivitis sicca) occurs in cases of inadequate tear volume or function, which results in an unstable tear film with disease of the surface of the eye. The prevalence of dry eye syndrome in American and Australian population is estimated to be around 5–16%, while in Asia it is higher and could reach up to 27–33% [49]. Severity of the symptoms may highly vary from patient to patient, but they mostly include a feeling of burning and blurred vision. Pathologies affecting the lacrimal gland and dysfunction of the Meibomian glands, as well as neurological diseases, can lead to the keratoconjunctivitis sicca [22,32]. The aim of therapy is to restore the normal surface of the eye by using tear supplementation along with inhibiting the aberrant inflammation observed in patients with chronic dry eye syndrome. Treatment of this condition is complex, but only the most common topical ocular formulations are discussed in this paper. The following groups of medicines are recommended: lubricants (carboxymethylcellulose, hydroxypropyl methylcellulose, and carbomer gels), hydroxypropyl guar/sodium hyaluronate or combinations (carboxymethylcellulose, polysaccharides or disaccharide), xanthan gum, phospholipids and soybean), artificial tears (with various electrolyte compositions, viscosity, and presence of preservatives), and eye drops with cyclosporine A (increasing tear production by reducing the inflammation of the lacrimal gland). However, lifitegrast is a topical anti-inflammatory medicine which is, at the moment, the only drug approved by the Food and Drug Administration for treating both symptoms and signs in patients with dry eye disease [48,49].

Cataract is a condition in which opacification of the crystalline lens occurs principally as a result of protein aggregation. According to some studies, the number of people in the world who become blind due to cataract is estimated as 20 million [52]. The Vision Loss Expert Group funded by the Bill and Melinda Gates Foundation, Fight for Sight, and others calculated that cataracts led to blindness in 10.6 million people and moderate to severe visual impairment in 34.4 million people [49]. A few classifications are used (depending on morphology, etiology, and maturity) but, in general, all cataracts can be divided into two groups: congenital and acquired. Among the latter group, one of the common types is the age-related cataract induced by metabolic processes of aging (including oxidative stress) of the human lens. Clinicians can hear from patients such symptoms as decreased visual acuity, changing of the contrast sensitivity, color perception, glare, monocular diplopia, and ghosting [48]. Surgical removal of the natural lens and its replacement with an intraocular lens (IOL) is currently the only solution for patients with this condition. However, scientists are actively searching for a potential topical treatment. Thus, it was found that pyruvate eye drops can effectively penetrate ocular membranes and potentially provide protection against oxidative stress [53], while Zhao et al. reported that using lanosterol-loaded nanoparticles can reverse protein aggregation in the lens [54]. 

Glaucoma is a progressive optic neuropathy that is accompanied by the excavation of the optic nerve head and a loss of visual sensitivity in the sequence beginning in the mid-peripheral visual field. Types of glaucoma are classified as open-angle, angle-closure, glaucoma due to another disease, and childhood onset glaucoma [55,56]. It is worth noting that the leading cause of irreversible blindness worldwide is glaucoma. The following indicators are considered as risk factors for developing glaucoma: increasing age (mostly after 40 years), race, family history, and using steroids. One of the major risks for the development and progression of glaucoma is intraocular pressure (IOP). The prevalence of this condition is 3.54% for people aged 40–80 years worldwide [56]. Even in normal-tension glaucoma with IOP not exceeding 21 mmHg, IOP remains a risk factor for progressive damage of the optic nerve [49]. One of the reasons why patients may not ask for medical help until developed stages of the disease occur is that it is usually asymptomatic. In some cases, patients may complain of halos, pain in the eye, headache, precipitants, and subjective loss of vision field [32]. Pharmacological treatment of glaucoma includes five main groups of topical formulations (prostaglandin analogues, beta-blockers, sympathomimetics (alpha-2-agonists), carbonic anhydrase inhibitors, and miotics). Eye drops with prostaglandin analogues (latanoprost, travoprost, bimatoprost, tafluprost) are considered to be a first-line therapy in open-angle glaucoma patients to reduce the IOP primarily by increasing uveoscleral outflow. A number of side effects (conjunctival hyperemia, irreversible hyperpigmentation of iris, reversible increasing pigmentation of the lid skin, lengthening along with thickening of eyelashes, and orbital fat loss), as well as limitations of using these therapies in inflamed eyes and in patients with herpetic keratitis in their anamnesis, may lead to replacing prostaglandin derivatives with beta-blockers. The latter IOP-lowering class of drugs reduces aqueous production and can be divided into two kinds of beta-blockers depending on the involved receptors: non-selective (timolol, carteolol, levobunolol) and β1-selective (betaxolol). However, the use of beta-blockers is relatively restricted in patients with asthma and cardiovascular diseases due to the potential bronchospasm, hypotension, heart block, and bradycardia. The carbonic anhydrase inhibitors (brinzolamide, dorzolamide) represent another type of topical formulations, which also lower levels of aqueous humor production, being contraindicated in patients with an allergy to sulfonamide antibiotics and patients with renal or liver failure. The mode of action of sympathomimetics (brimonidine and apraclonidine) is based on the stimulation of alpha-2-receptors, resulting in both the decrease of the aqueous secretion and enhancement of the uveoscleral outflow. On the other hand, bradycardia and heart block are among the contraindications for the use of these sympathomimetics. Alpha-2 agonists are mostly prescribed for short-term use (for instance, after laser iridotomy). The treatment of angle-closure glaucoma includes the use of miotics (pilocarpine and carbachol) that increase the outflow through the trabecular meshwork. Currently, numerous combined preparations are commercially available on the market (timolol and dorzolamide/brinzolamide/travoprost/bimatoprost/brimonidine/pilocarpine or brimonidine and brinzolamide). Regardless of the number of available options for topical treatment of glaucoma, there is still a demand of using systemic drugs, and laser and surgical procedures in some clinical cases [22,48].

Age-related macular degeneration (AMD) is a degenerative disorder affecting people over the age of 50 years. An estimated number of people affected by AMD is approximately 30–50 million worldwide [49]. There are two known forms of AMD. The majority of patients have so-called “dry” AMD that is usually asymptomatic, except for gradual central visual loss of night vision. Also, it may be accompanied by metamorphopsia and prolonged afterimages [57]. This stage is defined by formation of drusen (amorphous deposits located between the retinal pigmented epithelium (RPE) and the Bruch’s membrane) and abnormalities of RPE including hyperpigmentation and atrophy. In a relatively small share of patients, this form progresses to the neovascular (“exudative” or “wet”) AMD. This condition is characterized by the growth of new abnormal capillaries from the choriocapillaris (choroidal neovascularization) that penetrate the Bruch’s membrane, resulting in hemorrhages or exudation, producing scar, retina, or RPE detachment. At this stage, patients complain of a rapid onset of visual loss, a central blind spot, or metamorphopsia. The treatment of “dry” AMD is mostly based on changing lifestyle and using oral vitamin supplements (ascorbic acid, vitamin E, alpha-carotene, and zinc), which are thought to delay its progression. On the other hand, the treatment of the neovascular (“wet”) AMD should be started with intravitreal injections of anti-vascular endothelial growth factor agents (anti-vascular endothelial growth factor (VEGF) treatment: ranibizumab, aflibercept, pegaptanib, bevacizumab), which is an invasive procedure posing concern and discomfort for the patient. Moreover, there are some potential vision-threatening complications associated with intravitreal injections: infectious endophthalmitis, retinal tears, sterile inflammation, vitreous hemorrhage, cataract, elevation of the IOP, etc. [49]. The intravitreal injection still remains a primary delivery route of anti-VEGF agents. A serious limitation of this therapy is a relatively short half-life of VEGF following intravitreous injections [58], which implies the need for frequent administrations. Some approaches were recently reported to develop less invasive methods of delivery, for example, subconjunctival administration of lyophilized matrices containing bevacizumab [59].

The development of formulations capable of delivering anti-VEGF agents into the eye, when administered topically, will be of great advantage and could revolutionize the therapy of this condition. Davis et al. [60] reported the successful topical delivery of Avastin using annexin A5-associated liposomal formulations to the posterior segment of the eye in vivo (rats and rabbits) at physiologically significant levels. This could be considered as a major advancement that should attract further research and perhaps some studies into the use of penetration enhancers. Some advances in the development of topical formulations to the delivery of biopharmaceuticals to the posterior segment of the eye were recently discussed in several reviews [61]. 

Photodynamic therapy (PDT) is an option if patients have any contraindications for anti-VEGF therapy. Focal laser photocoagulation is the third solution that can be offered to a patient, which is uncommon due to common recurrence and presence of localized scotoma after the procedure [48]. 

Diabetic retinopathy (DR) also belongs to one of the leading causes of vision impairment, as prevalence of diabetes mellitus is increasing dramatically worldwide [62]. In fact, this is the most frequent microvascular complication of diabetes. According to Duh et al. [63], almost 100 million individuals were diagnosed with diabetic retinopathy. DR is divided into two groups: (1) the earlier stage of non-proliferative diabetic retinopathy (NPDR), which is characterized by microaneurysms, retinal hemorrhages, intraretinal microvascular abnormalities, and venous caliber changes; and (2) proliferative diabetic retinopathy (PDR) with pathologic pre-retinal neovascularization. Additionally, diabetic macular edema may occur during both NPDR and PDR, representing the most common cause of vision loss in patients with DR. This edema arises from diabetes-induced breakdown of the blood–retinal barrier, with consequent leakage of fluid and circulating proteins from the vessels into the neurosensory retina [63]. As a matter of priority, assessment of patients with DR relies on the control of glucose concentration in the patient’s blood. The treatment of this condition involves laser photocoagulation (panretinal, focal, and grid), intravitreal anti-VEGF agents (aflibercept and ranibizumab), corticosteroids (dexamethasone and fluocinolone acetonide intravitreal implants), and vitrectomy in the case of vitreous hemorrhage [48]. Some of these therapeutic approaches could potentially benefit from the development of formulations that could deliver drugs to the eye topically.

## 4. Permeability of Ocular Membranes

The analysis of the physicochemical properties of various chemical compounds that cross membranes in the eye could be key in understanding the opportunities and obstacles in ocular drug delivery. Thus, according to Prausnitz and Noonan [64], the octanol–water partitioning coefficient (logP), which helps characterize the drug’s hydrophilic–lipophilic properties, determines the ability of molecules to pass through cells of the epithelial and endothelial layers of the cornea and conjunctiva. The epithelium permeability accounts up to 90% for the lipophilic substances as a result of the high dependence on logP, while almost totally excluding macromolecules (with radius larger than 10 Å) [64]. Hence, the epithelium is the main limitation for intracorneal drug delivery. In contrast, stromal drug permeability is less dependent on logP but highly dependent on the radius of the molecules, providing a great barrier for lipophilic compounds of a small size (radius < 10 Å). Interestingly, the permeation across the endothelium layer relies on both logP and the size of the molecule (paracellular penetration route) and is slightly more impermeable for lipophilic small molecules in comparison to the corneal stroma. However, macromolecules cross the endothelium more easily than the stroma. The permeability of another ocular membrane, sclera, is relatively close to that of the corneal stroma. The data on conjunctival permeation are limited; however, according to some reports, it demonstrates a higher permeability compared to the cornea [64].

Edwards and Prausnitz [65] reported the development of a theoretical model that can help in predicting the permeability of the cornea for different solutes, which can be calculated using only two parameters: radius of the molecule and logP. This could be a very useful model in ocular drug delivery, but there are a few factors that were not taken into account by the authors: simplification of the structure of openings between the cells, as well as tight junctions in epithelial and endothelial layers, various permeability of cells within the epithelium layer, possible intracorneal binding of compounds, and different shape of molecules (assuming all of them to be solid spheres).

Recently, a few in vitro and ex vivo models were used to evaluate permeability and drug absorption by the cornea. Thus, according to Agarwal and Rupenthal [66], cell-based models are commonly used for studies of penetration. Advantages of these models include relatively lower cost compared to the use of laboratory animals, as well as minimizing the number of animal studies. However, this type of model is more suitable to evaluate the cytotoxicity of the compounds rather than their permeability and absorption by the cornea. This is because cell-based models are, in simple terms, five to six layers of the epithelial cells of the cornea (usually, corneal culture of the rabbit) but not the entire structure of the cornea. Additionally, these cell cultures neither have transporter molecules nor enzymes responsible for drugs metabolism. One of the closest models to the real cornea involves reconstructed tissue cultures which comprise different types of cells that help mimic the three-layer structure including the epithelium, stroma, and endothelium. For example, Kaluzhny et al. [67] recently developed an in vitro human three-dimensional corneal epithelial tissue model and demonstrated the applicability of this membrane for studies of drug permeability using several fluorescent markers, as well as latanoprost and bimatoprost.

**Figure 6:**
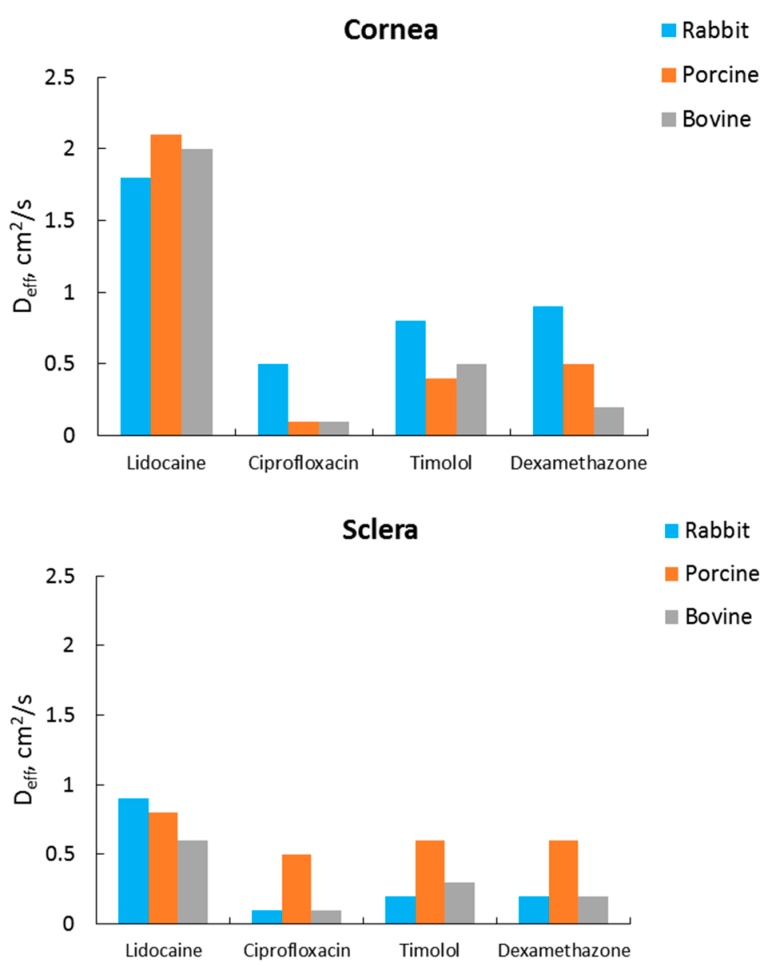
Effective diffusion coefficients *D_eff_* (cm^2^/s) of several drugs for rabbit, porcine, and bovine cornea and sclera. Data taken from Reference [21] with permission from Elsevier,2012.

Ex vivo corneas of various animals (rabbit, porcine, bovine) are also commonly used for evaluating corneal permeability and absorption; however, this model is not ideal due to the differences in the anatomical structure compared to the human cornea, as well as potentially different enzymes and molecules of active transporters. Thus, rabbit eyes are commonly used as ex vivo models, but the rabbit cornea does not have Bowman’s layer, which results in higher penetration of substances in comparison with the human cornea. The eyeball size, thickness of the cornea, ratio of length of the cornea to eye-globe diameter, and histological structure (including Bowman’s membrane) in porcine eyes are the closest to the human eye. Bovine eyes are also used in the studies of ocular tissue drug permeability despite the fact that these are larger than human eyes and their corneal epithelium is almost twice the thickness [66]. Loch et al. [21] conducted a comparative study of the permeation of three different drugs (ciprofloxacin hydrochloride, lidocaine hydrochloride, and timolol maleate) through porcine, rabbit, and bovine ocular tissues. They observed substantial differences between the apparent permeability coefficients (*P_app_*) for different animal species.

(1) $$P_{app}=\frac{Q}{A\times C_{0}},$$

where *Q* is the steady-state appearance rate of the investigated substance on the acceptor side of the tissue (mol/s), *C*_0_ is the initial concentration of the drug in the donor chamber (mol/L), and *A* is the surface area of the tissue (cm^2^). In order to take into account the differences in the thicknesses of porcine, rabbit, and bovine tissues, they calculated effective diffusion coefficients using the following equation:

(2) $$D_{eff}=\;P_{app}\times l,$$

where *l* is the tissue thickness (cm). The results of *D_eff_* determination are presented in Figure 6. These results clearly show that the chemical nature of the drug has great influence on the tissue permeability. The authors also hypothesized that protein binding, tissue hydration, or transporters may play some role in the differences between different species.

## 5. Penetration Enhancers

Anatomical and physiological features of the eye, ocular conditions, and permeability of ocular membranes were discussed above, and the review now moves to consider some of the different classes of penetration-enhancing compounds, describing their properties and modes of action.

### 5.1. Cyclodextrins

**Figure 7:**
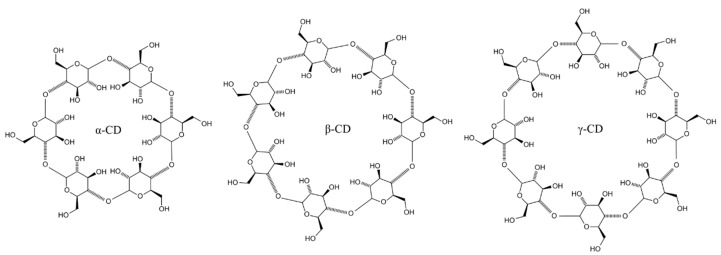
Structures of α-cyclodextrin, β-cyclodextrin, and γ-cyclodextrin. The image was reproduced under the Creative Commons Attribution Share Alike 3.0 Unported license [77].

Cyclodextrins (CD) are water-soluble cyclic oligosaccharides shaped like a truncated cone. Native cyclodextrins include α-CD, β-CD, and γ-CD, which differ in the number of α-(1-4)-linked glucopyranose subunits (six, seven, and eight, respectively, Figure 7) [68]. There were many derivatives of CDs developed over the last decades, each bringing some improved properties such as enhanced aqueous solubility. The most common derivatives of CDs include hydroxypropyl-β- and γ-CD, the randomly methylated β-CD, and sulfobutylether β-CD [69]. Cyclodextrins have lipophilic cavities and hydrophilic hydroxyl groups associated with the external surfaces of their molecule [70]. They are capable of forming guest–host inclusion complexes, whereby lipophilic drugs with poor aqueous solubility reside within the hydrophobic cavity, where they are protected, but not covalently bound. The drug–CD complexes usually have improved aqueous solubility due to the hydrophilic properties of the external surface of the cyclodextrin molecule [71]. It is generally established that only free drug molecules from topical formulations with cyclodextrins are able to penetrate ocular membranes [72]. Since cyclodextrins are relatively large molecules, they are unable to permeate through intact lipophilic membranes, for example, the corneal epithelium; however, they allow drugs to interact with the epithelial surface. They have uses in pharmaceutical applications due to their drug solubility-enhancing properties, improved bioavailability, and improved formulation stability, capable of masking a drug’s irritation effects; they are generally regarded as safe (GRAS) [73]. Drug–CD inclusion complex formation/dissociation is a dynamic process, where drug molecules are released and taken up spontaneously in the aqueous environment [74,75]. Lipophilic compounds are able to reside within the cyclodextrin molecule cavity through weak hydrophobic interactions. In the aqueous environment of the tear film, drugs can be released from drug–CD complexes by preferential take-up of cell-membrane lipids, such as cholesterol and phospholipids, and simultaneous ejection of the drug guest. There remains an opportunity for the drug to enter the epithelium membrane via the temporary disruption caused by lipid extraction during membrane–cyclodextrin interaction [68,76]. 

The ability of α-CD to enhance corneal penetration for cysteamine (β-mercaptoethylamine; medication used to treat cystinosis, which is a rare, genetic disorder with abnormal accumulation of cystine within the corneal stroma resulting in photophobia) [78] was established by Pescina et al. [79] in ex vivo experiments with freshly excised pig corneas. The researchers also demonstrated lack of irritation caused by a 5.5% α-CD solution employing the Hen’s Egg Test on the Chorioallantoic Membrane. Moreover, the addition of ethylenediamine-*N*,*N*,*N*’,*N*’-tetraacetic acid (EDTA) to α-CD established a good stability profile of cysteamine. According to Aktaş et al. [80], the eye drops consisting of pilocarpine nitrate and hydroxypropyl β-cyclodextrin (HP-β-CD) demonstrated a four-fold increase in transcorneal penetration compared to a drug formulation without CD. The researchers used side-by-side diffusion cells and corneas harvested from rabbits. In addition, the pupillary-response pattern was monitored in rabbits in vivo. The constriction of the pupil was remarkably increased due to the addition of the HP-β-CD-containing formulation of pilocarpine nitrate. Loftsson and Stefansson [81] designed low-viscosity eye-drop formulations with drug/γ-CD complexes which were then tested in vivo in rabbits and clinically in patients. The first aqueous eye-drop formulation included dorzolamide/γ-CD complexes and demonstrated high levels of the drug in the aqueous humor of rabbit eyes more than 24 h after a single application. The IOP-lowering effect of this solution in patients was observed after daily use compared to conventional eye drops with dorzolamide that should be given three times a day to provide the same effect. Other eye drops consisted of dexamethasone/γ-CD complexes and showed the ability to deliver dexamethasone to the posterior segment of the rabbit eye. Moreover, there were significant clinical improvements in patients with diabetic macular edema (DME) and intermediate uveitis patients with cystoid macular edema after the topical use of eye drops with dexamethasone/γ-CD. The results for DME patients were clinically similar to those after the intravitreal corticosteroid injection.

**Figure 8:**
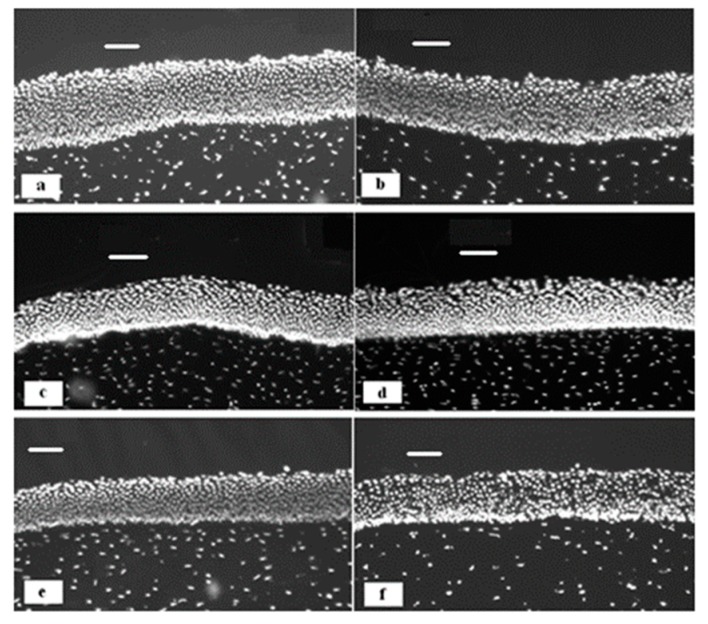
Micrographs of bovine cornea exposed to 1 mL of β-cyclodextrin (30 mg⋅mL^−1^) (**b**,**d**,**f**) against non-exposed regions (**a**,**c**,**e**). Exposure time: 15 (**a**,**b**), 45 (**c**,**d**), and 75 min (**e**,**f**). Scale bar = 100 µm. Reproduced from Reference [68] with permission from American Chemical Society, 2013.

Morrison and co-workers [68] studied the penetration of riboflavin through freshly excised bovine corneas using formulations containing various cyclodextrins. They established that β-CD and HP-β-CD facilitate the permeation of riboflavin through the cornea; however, they do not affect the lag time of approximately 90–120 min, where the first portions of the drug begin crossing the cornea. The microscopic examination of the corneas treated with cyclodextrins indicated that these permeability enhancers cause some disruption of the epithelial structure. Further analysis of this disruption allowed the authors to establish the mechanism of permeability enhancement related to the extraction of cholesterol from corneal tissue observed in the case of formulations with β-CD and HP-β-CD. This resulted in a partial disruption of the corneal epithelia, which was evidenced from histological examination of the bovine corneal membranes exposed to solutions of cyclodextrins for different contact times. Figure 8 shows exemplary micrographs of corneas exposed to 30 mg⋅mL^−1^ solutions of β-CD compared to control non-exposed tissues. Samples of the cornea treated with β-cyclodextrin showed some epithelial disruption, which became more noticeable with longer exposure time. However, no extraction of cholesterol was observed when the corneas were exposed to water or α-CD and γ-CD.

### 5.2. Chelating Agents

**Figure 9:**
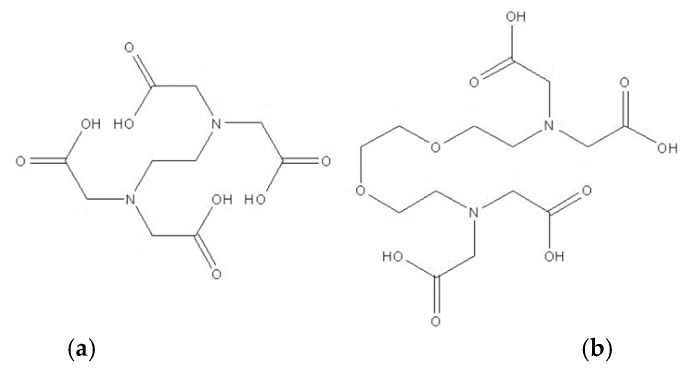

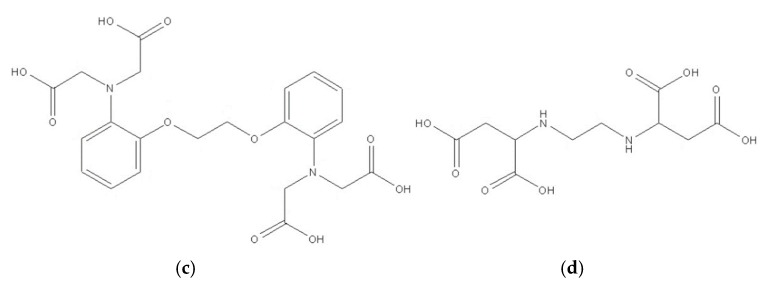
Structures of ethylenediamine-*N*,*N*,*N*’,*N*’-tetraacetic acid (EDTA) (**a**), ethylene glycol-bis(beta-aminoethyl)-*N*,*N*,*N*’,*N*’-tetraacetic acid (EGTA) (**b**), 1,2-bis(o-aminophenoxy)ethane-*N*,*N*,*N*’,*N*’-tetraacetic acid (BAPTA) (**c**), and ethylenediamine-*N*,*N*’-disuccinic acid (EDDS) (**d**).

The corneal epithelium offers a highly resistant barrier against alien matter due to its lipophilic characteristics, preventing the transcellular transport of many drugs, and the existence of tight junctions, i.e., tightly adherent regions between cells, maintaining intimate cell contact, effectively preventing drug transport via the paracellular route. Tight junction functionality of the superficial epithelial cells depends on an undetermined availability of Ca^2+^ ions [4,82]. Calcium chelating agents are often included in topical ocular drug formulations as stabilizers. They were shown to offer temporary and reversible action when used for enhancement of ocular drug delivery. However, there is evidence that ethylenediamine-*N*,*N*,*N*’,*N*’-tetraacetic acid (EDTA) in topical ocular formulations can bring side effects due to accumulation within the iris and ciliary body, and can affect endothelial cells and capillaries associated with the uveal tract. Caution should be exercised due to toxicity implications, especially when medication is required in the long term [83]. Calcium chelators, a class of polyaminocarboxylic acids, for example, EDTA, ethylene glycol-bis(beta-aminoethyl)-*N*,*N*,*N*’,*N*’-tetraacetic acid (EGTA), 1,2-bis(o-aminophenoxy)ethane-*N*,*N*,*N*’,*N*’-tetraacetic acid (BAPTA) [84], and ethylenediamine-*N*,*N*’-disuccinic acid (EDDS) (Figure 9), are capable of reversibly enhancing drug penetration across otherwise penetration-resistant barriers such as the corneal epithelium [17]. 

**Figure 10:**
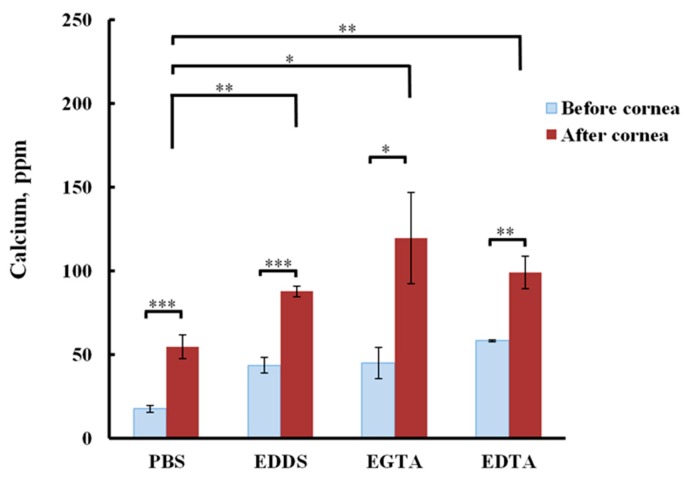
Calcium concentration in solutions containing phosphate-buffered saline (PBS), EDDS, EGTA, and EDTA (1 mg⋅mL^−1^) before and after 3 h of exposure to bovine cornea. * *p* < 0.05, ** *p* < 0.01, *** *p* < 0.001; one-way ANOVA; *n* = 3. Reproduced from Reference [17] under the terms of the Creative Commons Attribution License (CC BY).

Their mode of action is achieved via disruption of tight junctions and adherens junctions by sequestration of interstitial Ca^2+^ ions, on which the barrier function is dependent. Research carried out by Morrison and Khutoryanskiy [17] showed that low-concentration aqueous solutions of EDTA, EGTA, and EDDS at 1 mg⋅mL^−1^ were capable of extracting Ca^2+^ ions from bovine corneas in vitro (Figure 10). The three formulations studied enhanced Ca^2+^ extraction and corneal penetration of riboflavin compared with phosphate-buffered saline. The same researchers investigated permeability enhancement of bovine corneas in vitro using transepithelial electrical resistance analysis (TEER), finding that all polyaminocarboxylic acid formulations investigated lowered TEER values, correlating with enhanced penetration of riboflavin across bovine epithelia into the corneal stroma. EGTA showed the best performance for Ca^2+^ extraction and penetration enhancement from the formulations explored. 

Kikuchi et al. [85] investigated a synergistic relationship for enhancing ocular drug penetration when using a combination of EDTA and boric acid in various proportions. The researchers found that the co-formulation brought an improvement in drug penetration across the highly resistant epithelial membrane, but no enhancement to the already efficient permeation across de-epithelialized rabbit corneas. They concluded that the synergistic effects observed were due to improved transcellular permeability when compared to formulations with either EDTA or boric acid alone [85]. Topical aqueous ocular drug formulations incorporating polyaminocarboxylic acid penetration-enhancing agents induce disruption to the corneal epithelium, even at concentrations as low as 1 mg⋅mL^−1^. Although this enhances corneal permeability to drugs, it can also introduce undesirable side effects such as ocular irritation. The use of topical ocular drug formulations incorporating mucoadhesive polymers, i.e., hyaluronic acid, chitosan, and alginate, together with polyaminocarboxylic acid penetration-enhancing agents, gives the benefits from these chelating agents whilst moderating the epithelial disruptive effects, allowing them to give moderate enhancement to ocular drug penetration without inducing irritation. The use of mucoadhesive polymers potentially offers a means to give improved drug efficacy, low dose, and sustained delivery with reduced issues of toxicity and minimal undesired side effects [86,87]. However, Rodriguez et al. [86] demonstrated that the combination of mucoadhesive polymers with permeability enhancers leads to the inhibition of permeability-enhancing effects.

### 5.3. Crown Ethers

**Figure 11:**
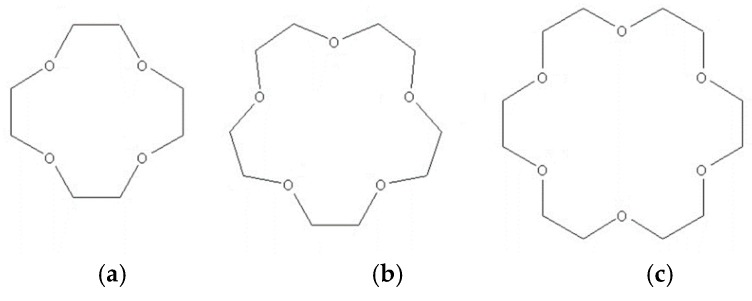
Structures of 12-crown-4 (**a**), 15-crown-5 (**b**), and 18-crown-6 (**c**).

Crown ethers are synthetic cyclic oligomers of ethylene oxide consisting of linked ether groups; they are named this way because the shape of their molecules resembles that of a crown when its structure is viewed at its side elevation. Naming convention follows numbers divided by the letter “C” for “crown”, whereby the larger number represents the number of atoms contained in the molecule, and the smaller number represents the number of oxygen atoms. The most common members are 12C4, 15C5, and 18C6, namely, the tetramer (*n* = 4), pentamer (*n* = 5), and hexamer (*n* = 6) (Figure 11).

Crown ethers were discovered accidentally by Pedersen whilst researching the development of complexing agents for divalent cations in the 1960s. During his research, Pedersen realized that the cyclic polyether by-products were capable of forming complexes with alkali metals [88]. Since Pedersen’s pioneering work, many derivatives were developed [89,90,91], and these found uses in various fields of science, industry, and pharmacology. Crown ethers are able to form complexes with metal ions, as well as neutral and ionic organic molecules, and their complexes have the ability to traverse biological membranes [92,93,94]. They are employed for the treatment of tumors [95,96] and drug-loaded vesicular preparations [97,98]. Crown ethers are flexible molecules, allowing them to adapt to the environment they are exposed to. In aqueous solutions, they interact with this medium by exposing their hydrophilic oxygen atoms to water molecules; however, when these molecules encounter a lipophilic solvent, they interact via their lipophilic ethylenic groups [91]. These properties, together with their ionophoric qualities, make them ideal compounds for use in ocular drug delivery, where the formulation has to interact with both aqueous and lipophilic phases. At the time of writing of this review, there was only one published study investigating the use of crown ethers for enhanced ocular drug delivery [99]; Morrison et al. [100] investigated 12C4, 15C5, and 18C6 for use in ocular drug delivery, and they were able to show enhanced aqueous solubility of riboflavin up to 46%, potentially improving bioavailability (Table 1). Furthermore, all crown ether variants studied were capable of significantly enhancing Ca^2+^ extraction from bovine corneas in vitro compared with phosphate-buffered saline. In the same study the researchers were able to show enhanced corneal delivery of riboflavin to bovine corneal stroma in vitro with crown ether concentrations as low as 1 mg⋅mL^−1^. 

Morrison et al. [100] also carried out a toxicological investigation and in vivo penetration studies using the rat model. The toxicological investigation was able to show that crown ethers were no more toxic to ocular tissue than benzalkonium chloride, an excipient often used at low concentrations as a preservative in ocular drug formulations. The in vivo drug penetration study failed to show any statistically significant permeability enhancement compared to enhancer-free formulations, the most probable reason being due to natural clearance mechanisms that protect the eye from foreign material. However, their in vivo study was conducted using a very limited number of animals (*n* = 3) to achieve good levels of statistical significance. They concluded that further in vitro and in vivo investigations are required to better understand crown ethers as penetration enhancers for ocular drug formulations [100].

### 5.4. Surfactants

Surface active compounds (surfactants) are compounds that have lipophilic and hydrophilic moieties. Acting on a surface, or between aqueous and non-aqueous media interfaces, surfactants are able to reduce interfacial surface tension [101]. In pharmaceutical applications, surfactants are commonly used as excipients to solubilize formulation components, or in the case of permeability enhancement, they alter membrane properties by disrupting tear film and mucin, removing their protective properties, disrupting the integrity of the epithelia by loosening tight junctions or modifying epithelial cell membranes. These compounds are included in formulations in a wide range of pharmaceutical applications including oral, injectable, nasal, ocular, and transdermal, amongst others. The polar group determines the specific properties of surfactants, and they can be classified into four main groups: cationic, anionic, zwitterionic, and non-ionic. Cationic surfactants have a positive charge on the polar head group, anionic surfactants have a negative charge, zwitterionic surfactants have positive and negative charges depending on which environment they are exposed to, and non-ionic surfactants are neutral [102]. Non-ionic surfactants are the compounds of choice for many drug delivery scenarios including ocular drug delivery, bringing enhanced drug solubility, formulation stability, biocompatibility, and low toxicity compared with their anionic, amphoteric, and cationic counterparts [103,104]. Polyoxyethylene-9-lauryl ether, Tween-80 and Span-60 are non-ionic surfactants, and their mode of action is via phospholipid acyl chain perturbation. According to Marsh et al. [105], the 1% solutions of Tween-20 and Brij-35 showed a relatively high effectiveness in enhancing permeability of the cornea by a five-fold increase in penetration of fluorescein through the cornea without detected irritation of the eye. Saettone et al. [106] reported improved permeability of the rabbit cornea for the β-blockers (atenolol, timolol, levobunolol, and betaxolol) combined with Brij-35, -78, and -98. The increased penetration was greater for atenolol and timolol, which are hydrophilic, than for hydrophobic β-blockers (levobunolol and betaxolol), which can be due to the hydrophilic properties of the corneal stroma. The non-ionic surfactant d-alpha-tocopheryl poly(ethylene glycol) 1000 succinate is also a potential permeability enhancer for ocular drug delivery. Ostacolo et al. [107] showed that this penetration enhancer effectively increased the permeation of riboflavin through porcine corneas. Pharmasolve^®^ (*N*-methyl-2-pyrrolidone) is another permeation enhancer that was studied by Li et al. [108] for transcorneal drug delivery using rabbit eyes. Interestingly, this penetration enhancer increased the apparent corneal permeability coefficients for ribavirin, enoxacin, puerarin, and ibuprofen by 4.04-, 2.76-, 2.67-, and 1.47-fold, respectively. Also, no ocular irritation was observed for formulations with less than 10% *N*-methyl-2-pyrrolidone. Montenegro et al. [109] demonstrated a significant improvement in penetration of acyclovir through the rabbit corneas in the presence of 5% *N*-methyl-2-pyrrolidone, positively charged phospholipid mixture, and sodium taurocholate after 90 min since the drug was administered on the ocular surface; however, after 180 min, only the phospholipid mixture was still effective. The taurocholate, Brij-78, and phospholipid mixture enhanced the permeation of timolol maleate across the cornea after 90 min, but only Brij-78 showed the ability to retain this effect after 180 min. 

**Figure 12:**
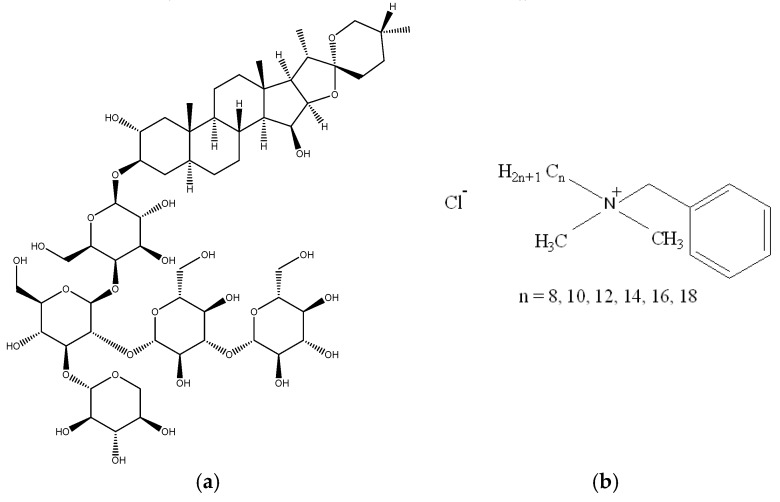
Structures of digitonin (**a**) and benzalkonium chloride (**b**).

These are also natural surfactants produced by some plants that could be potentially useful as penetration enhancers. Saponins are a class of amphiphilic glycosides abundantly found in different plants that exhibit good penetration-enhancement properties. For instance, digitonin is a steroidal saponin (Figure 12) isolated from the *Digitalis purpurea* plant; it is capable of solubilizing cellular membrane lipids and cholesterol. Shih and Lee demonstrated that digitonin caused ocular epithelial exfoliation [110,111,112]. 

Benzalkonium chloride (BAC) is a cationic surfactant that is commonly used in many formulations for ocular drugs in low concentrations as a preservative. BAC is a known irritant even at low concentration (<0.01%); nevertheless, it acts as a penetration enhancer, which destabilizes the tear film and the protection offered by the mucous layer at the cornea surface. BAC also initiates changes within phospholipid bilayers of cellular membranes [113]. Recently, Johannsdottir et al. [114] reported an in vivo study of benzalkonium chloride’s effect on the ocular penetration of dexamethasone in pigmented rabbits. They compared two eye-drop formulations of dexamethasone, where the drug formed a microsuspension with γ-CD in a vehicle containing poloxamer, NaCl, and EDTA with and without 0.02% (*w*/*v*) benzalkonium chloride. The analysis of the drug concentrations in different ocular tissues 2 h following the eye-drop administration indicated no statistically significant effects of benzalkonium chloride. The authors related the absence of permeability enhancement to the quick removal of benzalkonium chloride with tear fluid and its inability to penetrate into the cornea/conjunctiva and sclera to cause disruption of barrier function. 

Sasaki et al. [115] compared the influence of a few penetration enhancers (saponin, EDTA, benzalkonium chloride, and paraben) on improving ocular absorption of thyrotropin-releasing hormone and luteinizing hormone-releasing hormone using diffusion cells with rabbit conjunctiva and cornea. The researchers showed that penetration of both hormones through the conjunctiva was improved by 0.5% saponin, 0.05% benzalkonium chloride, and 0.01% benzalkonium chloride. The permeability coefficient of conjunctiva was significantly improved for luteinizing hormone-releasing hormone using EDTA and paraben. The observed effects of permeation enhancers for conjunctiva were smaller compared to those on corneal penetration with luteinizing hormone-releasing hormone with paraben as the exception. EDTA and saponin increased the ratios of corneal to conjunctival permeation of both hormones. According to van der Bijl et al. [116], 0.01% benzalkonium chloride enhanced the permeation of cyclosporin A through frozen/thawed rabbit cornea. 

Transcutol^®^ P is another solubilizer mostly used as a transdermal penetration enhancer. Liu et al. [117] demonstrated that 0.005–0.03% Transcutol^®^ P increased the apparent permeability coefficient by 1.5-, 1.5-, 3.0-, and 3.3-fold for ribavirin, gatifloxacin, levofloxacin hydrochloride, and enoxacin, respectively. Permeation for oxaprozin was inhibited with a maximum decrease by 2.8-fold for 0.03% Transcutol^®^ P. In addition, no irritation was observed with Transcutol^®^ P at concentrations of 0.005–0.03%, with the presence of slight irritation for 0.05% Transcutol^®^ P. A non-ionic oil-in-water surfactant, Labrasol^®^, is also a potential excipient as a corneal penetration enhancer. It is composed of well-characterized polyethylene glycol (PEG) esters, a small glyceride fraction, and free PEG. Liu et al. [118] observed no ocular surface irritation for 0.5–3.0% Labrasol^®^ and slight irritation for 5.0% Labrasol^®^. The penetration of baicalin solution through the rabbit cornea was enhanced by 1.69-, 3.14-, and 2.23-fold in the presence of 1.5%, 2.0%, and 3.0% Labrasol^®^, respectively. 

### 5.5. Bile Acids and Bile Salts

**Figure 13:**
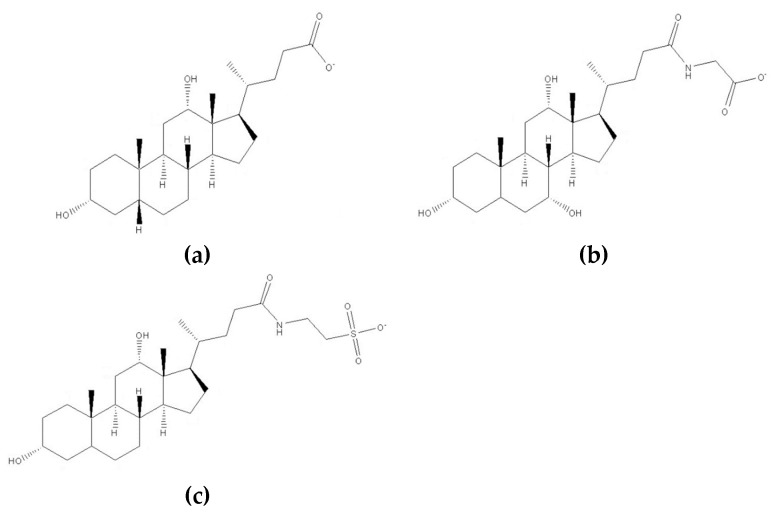
Structures of deoxycholate (**a**), glycocholate (**b**), and taurodeoxycholate (**c**).

Bile acids are steroid acids, all forms of cholic acid, and their sodium or potassium salts, and there are a number of derivatives that result from cholesterol metabolism in the liver which then undergo further transformations in the intestinal tract. They are soluble in water and are involved in the solubilization of dietary lipids within the gut. In the intestinal tract, they become conjugated with taurine and glycine, where the sodium and potassium salts of these conjugates are termed bile salts [119,120]. Bile acids are amphiphilic compounds naturally produced in the human digestive system; they are able to promote drug penetration across biological membranes. They are employed in drug formulations for enhancement of drug penetration via many mucosal routes including nasal, oral, buccal, ocular, pulmonary, and rectal mucosa. They are capable of forming vesicular drug-carrying systems which are able to promote epithelial drug transport via transcellular and paracellular routes [121]. Bile acids and bile salts have surfactant properties with an ability to form micelles or liposomes in the aqueous environment, otherwise known as “bilosomes”; these vesicular entities offer some interesting and novel properties in drug delivery [122]. Examples of many bile acid/bile salt derivatives include deoxycholate, glycocholate, and taurodeoxycholate (Figure 13). They are able to alter the rheological properties of ocular mucosal membranes [123], and the mucolytic properties of bile salts alter the protective mucus barrier, inducing changes in membrane characteristics allowing for enhanced drug transit into ocular tissue [4,124]. Dai et al. [125] investigated liposomes with bile salts for the ocular delivery of tacrolimus FK506, a calcineurin inhibitor, as a model drug. The liposomes they developed contained cholesterol (control) or bile salts (sodium taurocholate, sodium deoxycholate, and sodium glycocholate), prepared using a thin-membrane dispersion technique, achieving uniform particle size and drug entrapment. These liposomes exhibited controlled release of <5% over 24 h, thus avoiding a “burst release” often seen with some ocular drug delivery systems. They conducted ex vivo corneal permeability and in vivo corneal uptake experiments and demonstrated that liposomes with bile salts offered enhanced transcorneal drug permeability compared to the cholesterol-containing liposomes, enhancing the permeation of tacrolimus across the cornea up to four-fold. Furthermore, cytotoxicity studies showed that the liposomes with sodium taurocholate or sodium glycocholate were well tolerated, whereas those with deoxycholate showed evidence of corneal toxicity to spontaneously derived human corneal epithelial cells and in the rabbit model. The researchers concluded that liposomes incorporating sodium taurocholate and sodium glycocholate offer potential as ocular drug delivery systems for drugs of low aqueous solubility such as tacrolimus, due to their low toxicity and improved ocular permeability [125]. A cautious approach is generally required when employing bile acids and bile salts for enhanced ocular drug delivery; however, given the right choice of cholic acid variant or a derivative therewith, and the right choice of delivery vehicle, bile acids and their salts offer much potential for future ocular drug delivery systems.

Mahaling and Katti [126] recently investigated the effect of different enhancers (benzalkonium chloride, capric acid, EDTA, sodium glycocholate, and sodium taurocholate) on the penetration of polymeric nanoparticles in vivo in pigmented mice. It was established that sodium glycocholate and sodium taurocholate enhanced the permeability of nanoparticles in the conjunctiva. Benzalkonium chloride, sodium glycocholate, and sodium taurocholate enhanced bioavailability of nanoparticles in the iris and ciliary body. They also observed some inhibition effects; the use of EDTA decreased the bioavailability of nanoparticles in the lens and choroid, whereas sodium glycocholate showed a reduction in their presence in the choroid and retina. The authors concluded that a combination of penetration enhancers with a hydrophilic mucoadhesive coating on nanoparticles is a promising approach to enhance their bioavailability.

### 5.6. Cell-Penetrating Peptides

**Figure 14:**
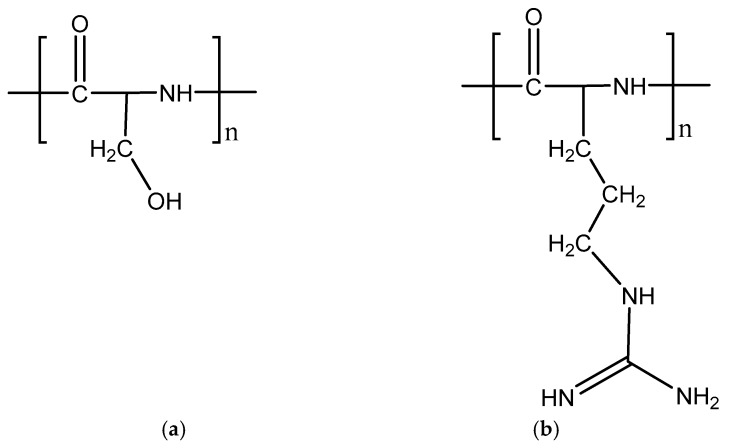
Structures of poly-l-serine (**a**) and poly-l-arginine hydrochloride (**b**).

Cell-penetrating peptides (CPPs) are short chains of amino acids connected by amide bonds (peptide bonds); they form a diverse group of compounds derived from variable combinations of many different natural amino acid residues [127]. CPPs are capable of penetrating cellular membranes and have the ability to transport internalized hydrophilic cargo into cells, for example, drugs [128]. Liu et al. [129] investigated a number of fluorophore-labeled cell-penetrating peptides, namely, trans-activating transcriptional activator (TAT), penetratin, poly(arginine), low-molecular-weight protamine, and poly(serine), for their ocular permeability using side-by-side diffusion cells, ex vivo in a rabbit model. Protamine is a small arginine-rich protein; TAT and penetratin are complex polypeptides consisting of specific sequences of different amino acids, whereas poly(arginine) and poly(serine) represent homopolymers of corresponding amino acids. The structures of poly(arginine) and poly(serine) are shown in Figure 14. They also used human conjunctival epithelial cells to determine cytotoxicity and cellular uptake of CPPs. The researchers found penetratin to have excellent performance in enhancing drug permeability, whilst also showing the lowest cytotoxicity. Penetratin showed cellular uptake more than 25-fold compared with the control peptide poly(serine). This peptide also showed an increased permeation of 87.5 times using the rabbit excised corneas. When fluorophore-labeled penetratin was instilled into the cul-de-sac of rat eyes, it was widely distributed in the anterior and posterior segments and could be measured in the corneal epithelium and retina for at least 6 h. They concluded that penetratin could be a potential permeability enhancer for ophthalmic use; this peptide can be conjugated with bioactive compounds for topical delivery into the eye reaching as far as the retina. Further research on the design and synthesis of new cell-penetrating peptides for topical delivery into different layers of the cornea, with potential application as absorption enhancers for metabolic sensitive ophthalmic drugs, was recently conducted by Pescina et al. [130]. The researchers labeled the synthesized CPPs with 5-carboxyfluorescein and measured their diffusion and distribution within the cornea using an ex vivo porcine model and confocal microscopy. The synthesized peptides were also shown to be safe and well tolerated when tested on human conjunctival cell line. They concluded that the tested CPPs could provide useful ocular therapies, especially when used as transcorneal transporters for some drugs with unfavorable molecular characteristics, examples being the aminoglycoside antibiotic, cysteamine, and antiviral agents. Interestingly, in 2017, de Cogan et al. [131] reported a promising approach for topical delivery of an anti-VEGF agent (bevacizumab) linked to the CPP (5(6)-carboxyfluorescein–RRRRRR–COOH) to the posterior segment of the eye. The toxicity of CPP, assessed using cell cultures, was found to be low. This complex study was done in vivo and included optical coherence tomography (OCT) imaging of the CPP tagged with fluorescent dye passing into the rat’s eye anterior segment, as well as assessment of topical delivery of the complex CPP–bevacizumab into the vitreous body with subsequent fixation of the time course of bevacizumab clearance from the vitreous body and retina. Moreover, the in vivo effectiveness of eye drops comprising a complex formulation of CPP with anti-VEGF agent was evaluated in a mouse model with choroidal neovascularization (CNV) which, as mentioned above, is common in patients with neovascular AMD. Mice received different treatment, which included intravitreal injection of the anti-VEGF agent, topical application of CPP with anti-VEGF (twice a day), or dexamethasone gavage (every day) for 10 days. Additionally, ex vivo experiments with topical delivery of anti-VEGF agents (ranibizumab and bevacizumab) into pig eyes were also conducted. As a result, CPP was observed within 6 min in the anterior chamber of the eye in rats. A single application of CPP–bevacizumab complex on the rat’s cornea showed drug levels in the posterior segment that could be relevant to clinical concentrations. CPP–ranibizumab and CPP–bevacizumab complex concentrations in the posterior chamber of porcine eyes were also found to be clinically relevant. There was a significant decrease in CNV area in all mice that received intravitreal injection, eye drops with CPP with anti-VEGF drug, and dexamethasone gavage compared to the eyes treated with a laser only. 

Nemoto et al. [132] demonstrated the permeability-enhancing features of poly-l-arginine hydrochloride for hydrophilic molecules (fluorescein isothiocyanate (FITC)-labeled dextran and pyridoxamine), across ocular membranes taken from Japanese white rabbits. The increase in *P_app_* of FITC-labeled dextran in the cornea, conjunctiva, and conjunctiva/sclera composite was observed in the presence of poly-l-arginine (0.1 mg/mL) by 6.81-, 9.78-, and 7.91-fold, respectively. The permeation of pyridoxamine was also improved by adding poly-l-arginine by 7.98-, 4.67-, and 8.31-fold, respectively. The authors suggested that the mechanism of this permeability enhancement lies in the ability of poly-l-arginine to disassemble tight junction-associated proteins present in the cornea and conjunctiva. Thus, this compound can be used as a permeability enhancer for lipophobic compounds without producing significant damage to the epithelium.

Some progress in the application of cell-penetrating peptides in ocular drug delivery was recently reviewed by Pescina et al. [133]. The authors highlighted this topic as an emerging area in ocular therapeutics, which has great potential for the delivery of drugs and biopharmaceuticals both to the anterior and posterior segments of the eye.

### 5.7. Other Amphiphilic Compounds

**Figure 15:**
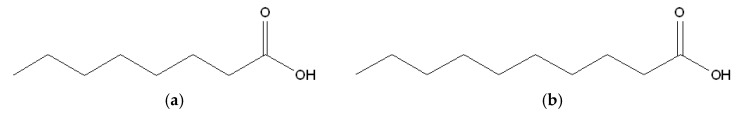
Structures of caprylic acid (**a**) and capric acid (**b**).

Fatty acids facilitate ocular drug permeation by altering cell-membrane properties and loosening tight junctions. These compounds can also induce ion–pair complexation when formulated with cationic drugs. Caprylic acid and capric acid are examples of fatty acids (Figure 15). The former interacts with proteins, whilst the latter can affect both proteins and lipid components of cellular membranes [111]. Capric acid was shown to enhance ocular penetration of β-blockers, bringing moderate enhancement to the penetration of hydrophilic β-blockers, whilst only offering slight enhancement for lipophilic β-blockers [123]. Kato and Iwata attributed the penetration-enhancing effects of fatty acids for bunazosin to ion–pair interactions [134,135].

**Figure 16:**
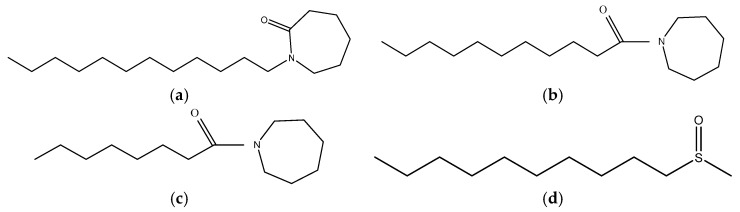
Structures of Azone™ (**a**), hexamethylenelauramide (**b**), hexamethyleneoctanamide (**c**), and decylmethylsulfoxide (**d**).

Gelucires are glyceride-based compounds with amphiphilic surfactant properties. Gelucires comprise mono-, di-, and triglycerides with mono- and diesters of polyethylene glycol [136]. The numbers 44 and 14 in the name of Gelucire 44/14 indicate the melting temperature of 44 °C and a hydrophilic–lipophilic balance of 14. Known for their drug absorption-enhancing performance, Gelucires have a good safety profile for pharmaceutical formulations and are “generally regarded as safe” (GRAS). Gelucire 44/14 was evaluated as a potential permeability enhancer in vitro and in vivo using different ophthalmic drugs and was shown to enhance transcorneal permeability of drugs with a range of hydrophilicity/lipophilicity whilst remaining safe to use and non-irritating [5,20]. Another potential corneal permeability enhancer is Azone™ (1-dodecylazacycloheptan-2-one), which is currently used mostly for transdermal drug delivery. This formulation is thought to act by partitioning into lipid bilayers of the bio-membrane and, as a result, disrupting its structure. Tang-Liu et al. [137] compared effects of four permeation enhancers (Azone™, hexamethylenelauramide, hexamethyleneoctanamide, and decylmethylsulfoxide (Figure 16)) with lipophobic (acetazolamide, cimetidine, guanethidine, and sulfacetamide), moderately hydrophobic (bunolol and prednisolone), and hydrophobic (flurbiprofen and its amide analogue) drugs through the rabbit cornea. 

The researchers demonstrated that 0.1% Azone™ enhanced the permeability of lipophobic formulations 20-fold, while permeation of moderately hydrophobic compounds was enhanced at 0.025–0.1% Azone™ by two- to five-fold. Interestingly, there was inhibition instead of promotion of corneal permeability for flurbiprofen and its amide analogue in the presence of Azone™. Also, all four penetration enhancers used in this study showed similar ability to promote penetration through the cornea for cimetidine. Additionally, the researchers noticed a parabolic relationship between the rate of drug penetration and the lipophilicity of this compound. Afouna et al. [138] also tested gel formulations with Azone™ as a permeability enhancer, Carbopol-974^®^ as a mucoadhesive, and *s*-timolol maleate as a model drug using rabbits. It was demonstrated that in vivo reduction of intraocular pressure for these formulations lasted roughly 3–4 times longer in comparison with the conventional timolol maleate eye drops. In 2016, Afouna et al. [139] reported the extension of in vitro parameters including release, onset, magnitude, and action duration up to two days for the gel formulations containing Azone™, Carbopol-974^®^, and latanoprost acid. 

**Figure 17:**
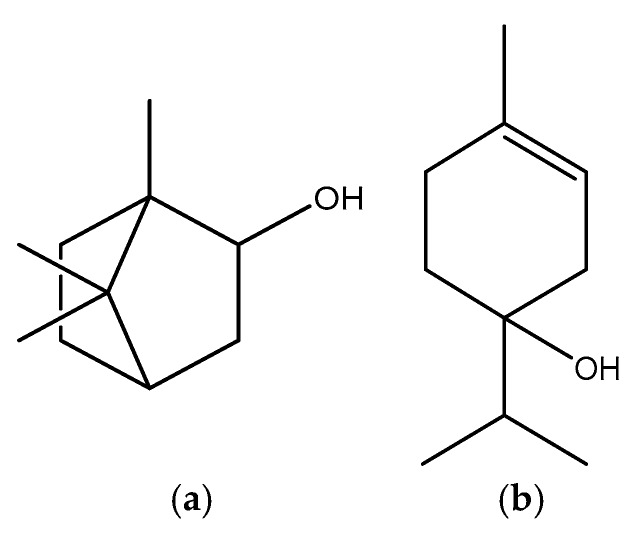
Structures of borneol (**a**) and terpinen-4-ol (**b**).

Borneol is a terpene derivative that can also be used as ocular penetration enhancer (Figure 17). According to Yang et al. [140], 0.1% synthetic borneol improved the permeability of two hydrophobic compounds (indomethacin and dexamethasone) through rabbit corneas by 1.23-and 2.40-fold, respectively, while the permeation of more hydrophilic drugs (ofloxacin, ribavirin, and tobramycin) was increased by 1.87-, 2.80-, and 3.89-fold, respectively. Natural borneol also enhanced the permeability with the following levels: 1.67, 2.00, 2.15, 2.18, and 3.39, respectively. It was also established that 0.1% borneol did not produce any damage to the cornea. The authors suggested that borneol’s ability of promoting corneal permeability might be due to the changes in the arrangement of lipid molecules in the cell membrane of corneal epitheliocytes, increasing the orderliness of the molecular chains of lecithin.

**Figure 18:**
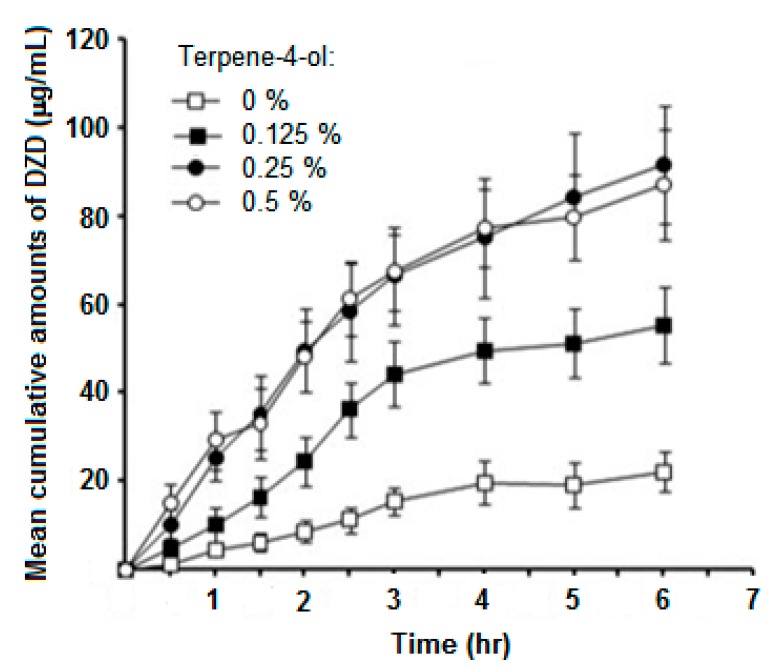
The cumulative amount of dorzolamide hydrochloride in the receiver chamber of a vertical Franz diffusion cell from ophthalmic gel formulations with various terpinen-4-ol concentrations and a fixed concentration of Carbopol-934 through the excised rabbit’s cornea (*n* = 3). Reproduced from Reference [141] with permission from Elsevier, 2010.

At the same time, terpinen-4-ol (Figure 17) can also be used as an ocular drug permeability enhancer. Afouna et al. [141] prepared and tested ophthalmic gel formulations with different concentrations of terpinen-4-ol as a penetration enhancer, Carbopol-934 as a mucoadhesive, and dorzolamide hydrochloride as a model drug. They demonstrated that permeation of this IOP-lowering drug across the excised rabbit’s cornea was increased significantly with the concentration of terpinen-4-ol. It was shown that the highest concentration of this penetration enhancer (0.5%) demonstrated the best permeation features among tested formulations. The authors suggested that this permeability improvement may result from the thermodynamic activity increase, as well as from the change in the ratio between ionized and unionized dorzolamide hydrochloride species in favor of the latter. The cumulative amount of dorzolamide hydrochloride in the receiver chamber of a vertical Franz diffusion cell from ophthalmic gel formulations with different terpinen-4-ol concentrations and fixed concentration of Carbopol-934 through the excised rabbit’s cornea is shown in Figure 18.

**Figure 19:**
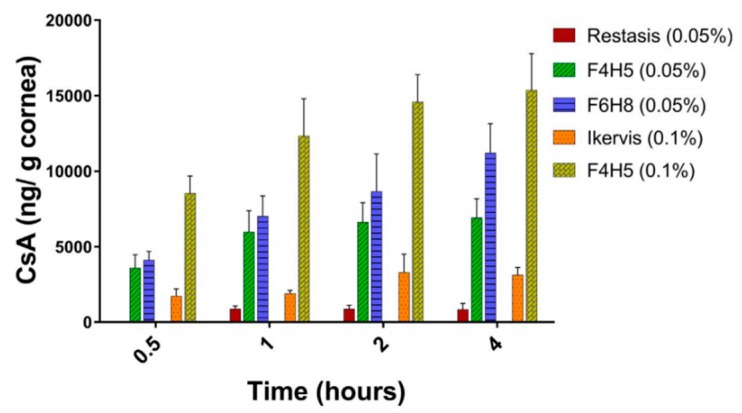
Corneal penetration of cyclosporin A (CsA) from the test formulations. The amount of CsA recovered (ng) per g of cornea after application of a single 50-µL dose was plotted against time (*n* = 5; mean ± standard error of the mean (SEM)). Reproduced from Reference [142] with permission from Elsevier, 2018.

Semifluorinated alkanes (SFAs) belong to the group of amphiphilic liquids that can dissolve lipophilic compounds forming clear solutions. Agarwal et al. [142] assessed the potential of two different SFAs for topical ocular drug delivery. Cyclosporin A (CsA) was dissolved in perfluorobutylpentane (F4H5) or perfluorohexyloctane (F6H8) and was compared with commercially available CsA ophthalmic emulsions, Restasis^®^ and Ikervis^®^, in terms of corneal availability. The penetration of CsA through the cornea was evaluated by plotting the concentration of the corneal CsA per g of cornea (ng/g) against time and determining the mean area under curve of each formulation tested over 4 h (AUC (0–4 h)) (Figure 19). The permeability of the cornea was significantly enhanced for CsA after a single dose of 0.05% CsA in F4H5 and F6H8 was applied when compared to Restasis with the area under the curve over 4 h (AUC (0–4 h)) being at least eight-fold higher for both SFAs. Interestingly, the AUC (0–4 h) of 0.1% CsA in F4H5 was almost five-fold higher than with Ikervis. Thus, semifluorinated alkane-based CsA formulations may improve the therapeutic efficacy. 

## 6. Comparison of Different Penetration Enhancers

Different classes of penetration enhancers considered in this review are summarized in Table 2. Some of these compounds, such as EDTA and benzalkonium chloride, are already commonly used in ophthalmic formulations with different roles, e.g., as a buffering agent and antimicrobial preservative, respectively. However, these compounds may provide some permeability enhancement as an extra benefit. Cyclodextrins are used in some pharmaceutical formulations to facilitate the solubility of poorly soluble drugs; additionally, they could provide permeability-enhancing properties. Other amphiphilic molecules such as bile acids and Azone^TM^ have established safety profiles but are not yet used in commercial ocular formulations. Crown ethers represent a relatively new class of permeability enhancers that will require more research into their efficiency and toxicological profile. Cell-penetrating peptides are highly promising permeability enhancers that received a lot of interest in the recent decade; more research is expected with these materials as they could potentially provide opportunities for formulating topical products for the delivery of biologicals. 

## 7. Conclusions

Topical drug application is the most widely used treatment in ophthalmology due to its simplicity. However, some obstacles including low permeability of the cornea, tear reflex, blinking, and nasolacrimal drainage hamper drug delivery in this way. The analysis of physicochemical properties of various chemical compounds that cross ocular membranes, coupled with the histological structure of cornea, sclera, and conjunctiva, could be key in understanding the opportunities and obstacles in the ocular drug delivery. Penetration enhancers facilitate delivery of active pharmaceutical compounds through three main mechanisms or their combination: altering tear film stability and the mucous layer at the ocular surface, modifying membrane components such as lipid bilayers of associated epithelial cells, and loosening epithelial tight junctions. The variety of penetration enhancers (cyclodextrins, chelating agents, crown ethers, bile acids and bile salts, cell-penetrating peptides, and other amphiphilic compounds) enables an increase in the permeability of ocular membranes. However, ocular drug delivery remains one of the toughest problems in ophthalmology, and new formulations still need to be developed to allow better control of drug delivery and improved performance, whilst also minimizing undesired side effects.

Different penetration enhancers were identified and mechanisms of their action were researched in the past several decades. Many of these enhancers were also evaluated for their potential harmful effects on the eye. However, these toxicological studies were often done to evaluate short-term exposure of the ocular tissues to enhancers. Very little is known on the longer-term exposure and potential chronic applications. 

## Figures and Tables

**Table 1:** Steady-state flux and apparent permeability of riboflavin through bovine cornea in phosphate-buffered saline (PBS) with and without crown ether. Reproduced from Reference [100] with permission from American Chemical Society, 2017.

Solution	Steady-State Flux (µmol∙min^−1^)	*P_app_* (cm∙s^−1^ × 10^−3^)
PBS	0.0478	1.945
12C4 (1 mg∙mL^−1^)	0.1208	6.138
15C5 (1 mg∙mL^−1^)	0.1074	5.457
18C6 (1 mg∙mL^−1^)	0.0753	3.826
12C4 (30 mg∙mL^−1^)	0.3410	17.326
15C5 (30 mg∙mL^−1^)	0.1961	9.964
18C6 (30 mg∙mL^−1^)	0.1811	9.202

**Table 2:** Different classes of penetration enhancers, their commercial applications, and possible mechanisms of action. EDTA—ethylenediamine-*N*,*N*,*N*’,*N*’-tetraacetic acid; TAT—trans-activating transcriptional activator; FDA—Food and Drug Administration.

Class of Enhancers	Examples of Compounds	Commercial Applications in Drug Delivery	Possible Mechanism of Penetration Enhancement
Cyclodextrins	α-, β-, γ-cyclodextrins	Some cyclodextrins are already used in commercial ocular formulations, e.g., Vitaseptol eye drops (Novartis). They are often used as enhancers of drug solubility.	Extraction of cholesterol and lipids from ocular membranes [68].
Chelating agents	EDTA	Disodium-EDTA is commonly used in ocular formulations as a buffering agent [143].	Extraction of Ca^2+^ from tight junctions [17].
Crown ethers	12-crown-4, 15-crown-5, 18-crown-6	None of these are currently used in commercial formulations for drug delivery.	Extraction of Ca^2+^ from tight junctions [100].
Surfactants	Benzalkonium chloride	Around 74% of ophthalmic preparations have benzalkonium chloride as a preservative [144].	Morphological changes in the epithelium [145].
Bile acids and salts	Deoxycholate, glycocholate, taurodeoxycholate	None of these are currently used in commercial formulations for drug delivery.	Different mechanisms leading to modification of the integrity of the corneal epithelium [121].
Cell-penetrating peptides	TAT, penetratin, poly(arginine), and poly(serine)	None of these are currently used in commercial formulations for drug delivery.	Direct translocation and endocytosis [133].
Other amphiphilic compounds	Azone™	Designed and widely researched mostly as a skin penetration enhancer. No FDA-approved products containing Azone^TM^ on the market yet. It is recorded in Chinese Pharmacopoeia and widely used in China [146,147].	Changes in the structure and fluidity of biological membranes; facilitation of water influx leading to a more hydrated barrier [137].
